# Lack of evidence to support the association of a single IL28B genotype SNP rs12979860 with the HTLV-1 clinical outcomes and proviral load

**DOI:** 10.1186/1471-2334-12-374

**Published:** 2012-12-23

**Authors:** Sabri Saeed Sanabani, Youko Nukui, Juliana Pereira, Antonio Charlys da Costa, Ana Carolina Soares de Oliveira, Rodrigo Pessôa, Fabio Eudes Leal, Aluisio C Segurado, Esper Georges Kallas, Ester Cerdeira Sabino

**Affiliations:** 1 Clinical Laboratory, Department of Pathology, LIM 03, Hospital das Clínicas (HC), School of Medicine, University of São Paulo, São Paulo, Brazil; 2 São Paulo Inistitute of Tropical Medicine, São Paulo, Brazil; 3 Department of Hematology, University of São Paulo, São Paulo, Brazil; 4 Division of Clinical Immunology and Allergy, University of Sao Paulo Medical School, São Paulo, Brazil; 5 Deparment of Infectious Diseases, School of Medicine, University of Sao Paulo, São Paulo, Brazil

**Keywords:** HTLV-1, ILB 28 polymorphisms, HAM/TSP, Proviral load

## Abstract

**Background:**

The Interleukin 28B (IL28B) rs12979860 polymorphisms was recently reported to be associated with the human T-cell leukemia virus type 1 (HTLV-1) proviral load (PvL) and the development of the HTLV-1-associated myelopathy/tropical spastic paraparesis (HAM/TSP).

**Methods:**

In an attempt to examine this hypothesis, we assessed the association of the rs12979860 genotypes with HTLV-1 PvL levels and clinical status in 112 unrelated Brazilian subjects (81 HTLV-1 asymptomatic carriers, 24 individuals with HAM/TSP and 7 with Adult T cell Leukemia/Lymphoma (ATLL)).

**Results:**

All 112 samples were successfully genotyped and their PvLs compared. Neither the homozygote TT nor the heterozygote CT mutations nor the combination genotypes (TT/CT) were associated with a greater PvL. We also observed no significant difference in allele distribution between asymptomatic carriers and patients with HTLV-1 associated HAM/TSP.

**Conclusions:**

Our study failed to support the previously reported positive association between the IL28B rs12979860 polymorphisms and an increased risk of developing HAM/TSP in the Brazilian population.

## Background

Human T-lymphotropic virus type I (HTLV-I) is an oncogenic human retrovirus that was first isolated from a T-cell line, HUT102, that had been obtained from a patient with adult T-cell leukemia/lymphoma (ATLL) [1]. Globally, there are an estimated 10–20 million individuals that carry the HTLV-I [2]. The disease burden is unevenly distributed, with a higher incidence of the disease particularly in southwest Japan, the Caribbean islands, South America, and portions of Central Africa [3]. Infection with HTLV-I may result in a spectrum of clinical manifestations, ranging from asymptomatic infection to a number of human disorders, most notably a malignant ATLL and a chronic progressive neuromyelopathy, termed HTLV-I-associated myelopathy/tropical spastic paraparesis (HAM/TSP) [4]. The majority of HTLV-I-infected individuals remain asymptomatic for life, while some individuals progress to a preleukemic phase that is characterized by small numbers of circulating leukemic cells in the peripheral blood, skin lesions, and a lack of involvement of other organ systems [5]. Only 2.5% to 5% of the virus carriers eventually develop ATLL after a long asymptomatic period [6,7]. The reason why some people develop disease, whereas others remain healthy, is likely dependent on both host-related and virus-related factors [8]. Available evidence from molecular studies indicate that the impairment of various cellular functions by viral genes (e.g., *tax* and *HBZ*), genetic and epigenetic changes including DNA methylation, and the host immune system may contribute to the leukemogenesis of ATLL [9-11].

Cytokines play an indispensable role in the defense against viral infection, both indirectly through determination of the predominant pattern of the host response and directly through significant inhibition of viral replication [12]. In attempts at explaining the heterogeneity of HTLV-I courses and outcomes, considerable attention in recent years has been paid to the single nucleotide polymorphisms (SNPs) in host genes that encode cytokine receptors and human leukocyte antigen (HLA) class I and II [13-19]. For example, multiple studies have suggested an association between polymorphisms at the IL-6 and IL-10 promoters, at position −634 and −592, respectively, and the pathogenesis of HAM/TSP [18,19]. In contrast, genetic variation at site −592 resulted in no association in other study analyzing the impact of these polymorphisms on the risk of HAM/TSP in Brazilian patients [20]. In another study, a 857 T polymorphism in tumor necrosis factor α (TNF-α), which is a potent immuno-modulator and pro-inflammatory cytokine, has been shown to be associated with ATLL patients compared to healthy subjects, suggesting that the genetic alteration that increases the production of TNF-α is associated with a susceptibility to ATLL [13].

Recently, three independent genome-wide association studies identified several polymorphisms, such as rs8099917, rs12979860, and 12980275, in or near the IL28B gene in patients infected with hepatitis C virus genotype 1 who had previously been treated with a combination of pegylated IFN-α and ribavirin [21-23]. The polymorphism rs12979860 (3 kb upstream of IL28B), identified by Ge et al*.,*[21] was described as the SNP with the strongest association to sustained virological response, which is defined as the undetectability of HCV RNA 6 months after stopping therapy. This breakthrough association has now been found in various studies worldwide [24-27]. Interestingly, HTLV-1 infected individuals with the CT/TT allelic variants at the IL28B rs12979860 gene exhibited approximately a 3-fold increased risk of HAM/TSP and had nearly 10-fold higher median HTLV-1 proviral loads (PvLs) than CC carriers [28]. To investigate the consistency of this association, we chose to further scrutinize the role of the IL28B rs12979860 polymorphism in both asymptomatic HTLV-1 carriers and in infected individuals that display clinical symptoms (*n =*112), in correlation to PvL.

## Methods

All patients were Brazilian and were recruited from the HTLV-1 outpatient clinic at the University of Sao Paulo, Brazil. We were unable to recruit more subjects, which would have enhanced the power of the study, and the total number of subjects was thus limited to 112. Thus, our study has an 80% power to detect a minimum odds ratio of 1.5 for the SNP, assuming α = 0.05 and the two tailed test.

Peripheral blood samples (5 ml) were collected from the 112 HTLV-1 positive individuals. Infected individuals were identified by the HTLV enzyme immunoassays Murex HTLV I + II (Abbott/Murex, Wiesbaden, Germany) and Vironostika HTLVI/II (bioMérieux bv, Boxtel, Netherlands), and infection was confirmed by HTLV BLOT 2.4 (HTLV blot 2.4, Genelabs Diagnostics, Science Park,. Singapore). Of these, 81 (72.3%) were asymptomatic carriers (ACs), 24 (21.4%) had HAM/TSP and 7 (6.3%) had ATLL. All ACs were diagnosed as HTLV-1 carriers at the time of blood donation. The clinical status of HAM/TSP was determined based on WHO criteria for HTLV-1 associated diseases [29]. Diagnostic criteria for ATLL included serologic evidence of HTLV-1 infection and cytologically or histologically proven T cell malignancy. Written informed consent was obtained from each participant. The study was approved by the local review board (Comissão de Ética para Análise de Projetos de Pesquisa, CAPPesq).

Laboratory measurements included peripheral blood lymphocyte counts, quantitative measurement of HTLV-1 PvLs with the use of sensitive SYBR Green Real-Time PCR with a detection limit of 1 copy per 10^3^ PBMC cells, and HTLV-1 genotypes [30]. The results were obtained from the patients' medical records.

Based on the published human chromosome 19q13 sequence (NCBI Reference Sequence: NT_011109.16), a 293 base pair segment upstream of the IL-28 gene, flanking the rs12979860 polymorphism between nucleotides 12006835 and 120071103, relative to the chromosome 19 translational start codon, was analyzed. This region was PCR-amplified from genomic DNA using the forward primer 5'-GCTCAGCGCCTCTTCCTCCTGCG-3' and the reverse primer 5'-GGCAGGGCTCCCTTCTGTGATTGACC-3'. The PCR conditions consisted of an initial denaturation step at 95°C for 3 minutes, followed by 35 cycles of 95°C for 30 seconds, 58°C for 30 seconds and 68°C for 1 minute, and a final extension at 68°C for 5 minutes. Negative controls without the DNA template were included in each experiment. After termination of the PCR cycle, the products were purified using a QIA quick PCR purification kit (Qiagen). Complementary DNA strands from each amplicon were directly sequenced by cycle sequencing using the same primers used for the PCR, BigDye terminator chemistry and *Taq* polymerase on an automated sequencer (ABI 3130, Applied Biosystems Inc., Foster City, CA), according to the protocols recommended by the manufacturer.

Continuous variables, including PvLs, were presented as the median (range) or mean ± standard deviation, while allelic frequencies were estimated by direct allele counting and were expressed as frequencies (%). Deviation from Hardy-Weinberg equilibrium was tested by a standard *χ*2 test with 1 degree of freedom. Differences between PvL variables were assessed by means of Student’s *t* test with the non-parametric U-Mann–Whitney test, while those between allelic frequencies variables were evaluated using the Pearson *χ*2 test with Yate's correction or Fisher’s exact test, when appropriate. A *p* value < 0.05 was considered significant*.* The data were analyzed with Stata statistical software (StataCorp, release 5.0, 1997; Stata, College Station, TX).

## Results

In total, 112 blood samples from HTLV-1 infected individuals were included in the study. The participants' ages ranged between 24 and 73 years, and the median age was 55 years. Females constituted 62.5% (*n* = 70) of the study population. The median measurements of CD4, CD8 and CD25 lymphocyte percentage by flow cytometry (FACScan, Becton-Dickinson, Cowley, Oxford) were 45%, 26%, and 22%, respectively, in 65% of subjects. The main characteristics of the study population are given in Table 1.

**Table 1:** Characteristics of the 112 study subjects

**Age. Years**	
Mean ± SD	51.9 ± 12.6
Median	55
Range	24-73
Gender (%)	
Male	42 (37.5)
Female	70 (72.5)
Median lymphocyte subpopulations	
% CD4 cells	45
% CD8 cells	26
% CD25 cells	22
Clinical status (%)	
Asymptomatic carriers	81 (72.3)
HAM/TSP	24 (21.4)
ATLL	7 (6.3)

**Figure 1:**
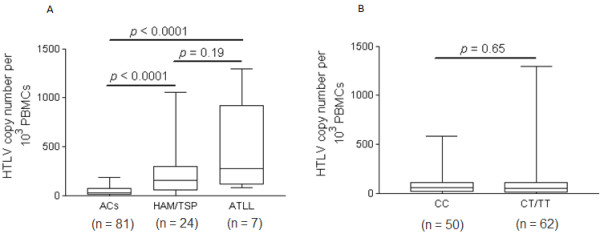
**Distribution of HTLV-1 proviral load levels among study subjects.** (**A**) Box and Whiskers plot of HTLV-1 proviral load in asymptomatic HTLV-1 carriers, HTLV-1–associated myelopathy/tropical spastic paraparesis patients and Adult T Cell Leukemia/Lymphoma. (**B**) Interleukin 28B CC versus CT/TT allelic variants.

The median PvL and the frequency of clinical status among subjects are shown in Figure 1A. The PvL levels were significantly higher in both HAM/TSP patients (median 60 copies/1000 PBMCs) and ATLL patients (median 272 copies/1000 PBMCs) (*p* < 0.0001, for each), compared to ACs.

In the entire group, the frequencies of the CC, CT and TT genotypes of rs12979860 were 45%, 48% and 7%, respectively, reflecting a C allele frequency of 93%. The calculated distribution of the rs12979860 alleles according to the Hardy-Weinberg equilibrium was 69% for the C allele and 31% for the T allele. Because of the small number of subjects who were homozygous for the TT allele (*n* = 7), and because PvL values in individuals with this genotype and in individuals that have the heterozygote CT genotype were not significantly different (*p* = 0.4), subjects that were homo- or heterozygotes for the T mutated allele (CT/TT) were grouped together for further analysis. Next, we analyzed the relationship between the PvL levels and the rs12979860 alleles. As shown in Figure 1B, the median HTLV-1 PvL levels did not differ significantly between subjects carrying the rs12979860 CC genotype (median 57.5 copies/1000 PBMCs) and patients with a CT genotype (median 48 copies/1000 PBMCs) or a TT genotype (median 53.5 copies/1000 PBMCs) (*p* = 0.7).

**Figure 2:**
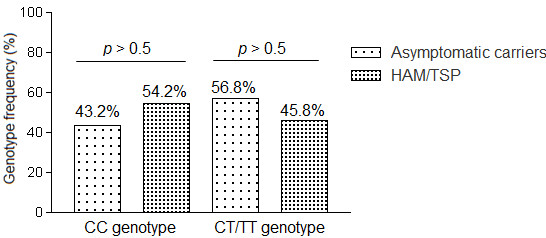
Comparison of the frequencies of interleukin (IL) 28B rs12979860 CC and CT/TT allelic variants between asymptomatic HTLV-1 carriers and HTLV-1–associated myelopathy/tropical spastic paraparesis patients.

We further aimed to analyze the data for potential correlates of clinical status and frequencies of the rs12979860 SNP. To this end, a comparison was made between patients with HAM/TSP and HTLV-1 ACs. Because of the small number of subjects (*n* = 7; 2 CC, 5 CT and 1 TT), ATLL patients were excluded from analysis. The frequency of the rs12979860 CC genotype was 43.2% among the 81 AC subjects and 54.2% among the 24 patients with HAM/TSP (*p* > 0.5). Similarly, the frequency of the CT/TT genotypes was not appreciably different between the two groups (*p* > 0.5; Figure 2).

The transcontinental subgroup A of HTLV-1a was the major subtype accounting for 90.4% of the studied subjects. No significant difference in the frequencies of the rs12979860 genotypes identified was found in this study, when HTLV-1 subtypes were compared.

## Discussion

In this study, we sought to replicate specific findings of association between the polymorphism rs12979860 in the vicinity of the IL28 gene and the HTLV-1 PvL, which is itself a predictive factor for development of HAM/TSP or ATLL [14,32-36]. However, our data reveal no support for association between this SNP and HTLV-1 proviral DNA levels, in Brazilian subjects, regardless of whether they are symptomatic or are ACs. The results also held true when the analysis was extended to compare the rs12979860 polymorphism frequencies in patients that have HAM/TSP, have ATLL or are asymptomatic carriers. Recently, Treviño et al. [28] have reported a significantly greater frequency of the CT/TT allelic variant in patients with HAM/TSP than in ACs (80% *vs.* 20%; *p* = 0.03), and a significantly higher median HTLV-1 PvL was found in individuals with the CT/TT variant than in those with the CC variant (*p* = 0.01). In contrast to this report, our data do not support the IL28B gene polymorphism as a novel genetic susceptibility marker, either for HTLV-1-associated HAM/TSP nor ATLL, despite involving more than twice as many cases of HAM/TSP as the recent study [28]. The reasons for this discrepancy remain unclear, but it is possible that the original associations for these SNP were either due to chance or were overestimated due to the small sample size. It seems highly unlikely that this inconsistency in findings may stem from different methods used to assign the rs12979860 polymorphisms. The initial study used an allelic discrimination fluorogenic probe, and we used direct sequencing approach, which is still considered to be the gold standard. We believe that these 2 methods are similar to each other, and we assume that there is no difference in the definition of the term “genotype” between the two studies. We acknowledge that our data must be interpreted cautiously, and we cannot rule out sampling bias that results from the small sample size of this study. However, we believe that if the findings of the recent study were not due to chance, they might generalize beyond the HTLV-1 infected subjects reported in that study. Unfortunately, our study provides no support for that hypothesis and argues that the association of the IL28B polymorphism and the risk of HAM/TSP be viewed with skepticism; many of the statistical associations between a disease and alleles of specific genes have been irreproducible in the past [37].

Several recent studies have also assessed the association of the IL28B rs12979860 SNP in other viral infections in which IFN plays a critical role, and the results of those studies were similar to ours. For instance, the study by Rallon *et al.*[38] found a nonsignificant association between IL28B polymorphisms and neither HIV disease progression nor HIV protection in a sample of 29 seronegative individuals at risk for HIV-infection and in 68 HIV-positive carriers either with or without rapid progression of immunodeficiency. Martin *et al*. [39] also found a nonsignificant association between IL28B SNP and recovery of hepatitis B virus (HBV) infection in a sample of 226 individuals with HBV persistence and 384 with HBV recovery. Furthermore, Scagnolari and colleagues [31] reported a lack of association between IL28B SNP and the clinical course of bronchiolitis caused by respiratory syncytial virus. Yet another recent study by Kamihira *et al.*[40] reported that the frequency of the rs8099917, located 3 kb upstream the IL28B/IFN-l3 gene [21], had no significant association with susceptibility to HTLV-1 infection or the development of ATLL in the Japanese subjects. Therefore, it is unlikely that the two SNPs (rs8099917 and rs12979860) are predictive factor for development of HAM/TSP or ATLL. Future studies are required to determine the extent to which differences in the ILB28 rs12980275 allelic variants frequencies between HTLV-1 infected subjects predict disease prevalence differences between populations.

## Conclusions

We observed no compelling evidence of an association between the IL28B allelic variants and HTLV-1 PvL. Additionally, we were unable to confirm the hypothesis that the IL28B CT/TT polymorphisms might be a genetic marker for HTLV-1-associated HAM/TSP risk. These findings do not support the clinical utility of either testing for the IL28B in all asymptomatic HTLV-1 individuals, or closer follow-up of IL28B CT/TT carriers, as these steps could put us at risk of diverting financial resources from research areas that might make more of a difference in our understanding of the etiological mechanisms of HTLV-1 associated disease.

## Competing interests

The authors declare that they have no competing interests.

## Authors’ contributions

SSS wrote the manuscript and directed the study. YN, JP, FEL, ACS and EGK were responsible of the clinical management of patients and acquisition of data, ACC, ACSO RP participated in the design of study, performed the experiments and the statistical analysis. FEL, EGK and ECS contributed to drafting the manuscript. All authors read and approved the final version of the manuscript.

## Pre-publication history

The pre-publication history for this paper can be accessed here:

http://www.biomedcentral.com/1471-2334/12/374/prepub
